# Long Time to Diagnosis of Medulloblastoma in Children Is Not Associated with Decreased Survival or with Worse Neurological Outcome

**DOI:** 10.1371/journal.pone.0033415

**Published:** 2012-04-02

**Authors:** Jean-Francois Brasme, Jacques Grill, Francois Doz, Brigitte Lacour, Dominique Valteau-Couanet, Stephan Gaillard, Olivier Delalande, Nozar Aghakhani, Stéphanie Puget, Martin Chalumeau

**Affiliations:** 1 Department of Pediatric and Adolescent Oncology, Institut Gustave Roussy, Villejuif, France; 2 Université Paris Sud, Le Kremlin Bicêtre, France; 3 Inserm U953, Epidemiological Research Unit on Perinatal Health and Women's and Children's Health, Hôpital Saint-Vincent-de-Paul, Paris, France; 4 Department of Pediatric and Adolescent Oncology, Institut Curie, Paris, France; 5 Université Paris Descartes, Paris, France; 6 French National Registry of Childhood Solid Tumors, CHU Nancy, Vandoeuvre-lès-Nancy, France; 7 Department of Neurosurgery, Hôpital Foch, Suresnes, France; 8 Université Paris Ile-de-France Ouest, Guyancourt, France; 9 Department of Pediatric Neurosurgery, Fondation Rothschild, Paris, France; 10 Department of Neurosurgery, Centre Hospitalier de Bicêtre, AP-HP, Le Kremlin Bicetre, France; 11 Department of Pediatric Neurosurgery, Hôpital Necker-Enfants Malades, AP-HP, Paris, France; 12 Department of Pediatrics, Hôpital Necker-Enfants Malades, AP-HP, Paris, France; University of York, United Kingdom

## Abstract

**Background:**

The long time to diagnosis of medulloblastoma, one of the most frequent brain tumors in children, is the source of painful remorse and sometimes lawsuits. We analyzed its consequences for tumor stage, survival, and sequelae.

**Patients and Methods:**

This retrospective population-based cohort study included all cases of pediatric medulloblastoma from a region of France between 1990 and 2005. We collected the demographic, clinical, and tumor data and analyzed the relations between the interval from symptom onset until diagnosis, initial disease stage, survival, and neuropsychological and neurological outcome.

**Results:**

The median interval from symptom onset until diagnosis for the 166 cases was 65 days (interquartile range 31–121, range 3–457). A long interval (defined as longer than the median) was associated with a lower frequency of metastasis in the univariate and multivariate analyses and with a larger tumor volume, desmoplastic histology, and longer survival in the univariate analysis, but not after adjustment for confounding factors. The time to diagnosis was significantly associated with IQ score among survivors. No significant relation was found between the time to diagnosis and neurological disability. In the 62 patients with metastases, a long prediagnosis interval was associated with a higher T stage, infiltration of the fourth ventricle floor, and incomplete surgical resection; it nonetheless did not influence survival significantly in this subgroup.

**Conclusions:**

We found complex and often inverse relations between time to diagnosis of medulloblastoma in children and initial severity factors, survival, and neuropsychological and neurological outcome. This interval appears due more to the nature of the tumor and its progression than to parental or medical factors. These conclusions should be taken into account in the information provided to parents and in expert assessments produced for malpractice claims.

## Introduction

Brain tumors are the leading cause of solid cancers in children [1]. Medulloblastoma, one of the most common types [1], has a 10-year survival rate of 50% [2]–[5], and many survivors have neurological and cognitive sequelae [6], [7]. The time to diagnosis for brain tumors is one of the longest of all childhood cancers, with a median ranging from 2 to 5 months [8]–[24]. We showed recently that the time from symptom onset to diagnosis of medulloblastoma is long (median 65 days), and we analyzed the causes of this lengthy interval. In particular, it was significantly associated with the presence of apparently psychological symptoms [25].

The delay in diagnosis of childhood tumors leads to painful remorse or guilt feelings for parents and physicians, loss of confidence and sometimes conflicts [26], [27]. In some countries it is a leading cause of pediatric malpractice suits [28]. Its precise consequences, however, have been little studied [18]. The obvious hypothesis is that the longer the tumor has to develop, the more extensive it will be (higher stage), and therefore the poorer the prognosis. Results thus far available in the literature tend to contradict this hypothesis. Only two studies are available for medulloblastoma specifically: one found no relation in either direction between delay and survival [17], while the other reported an inverse relation between duration of symptoms and metastasis [13]. Nonetheless several factors limit the usefulness of these results: the limited numbers (<100) of pediatric patients [13], [17], pooled analyses for pediatric and adult patients [13], single-center recruitment subject to selection bias [13], incomplete initial disease staging for some patients that increases the likelihood of classification bias [13], a study period partially preceding the availability of CT and MRI [13], and a lack of multivariate analyses despite the presence of potential confounders [13], [17]. Finally, none of these studies analyzed the relation between time to diagnosis and either local tumor stage, complete surgical resection or neurological and cognitive sequelae.

Our objective was therefore to analyze, in a pediatric population-based study, the consequences of the time to diagnosis of medulloblastoma on initial tumor stage, survival, and neuropsychological and neurological outcome, while taking confounding factors into account.

## Methods

### Patients

We conducted a multicenter historic population-based cohort study that included all patients in one French region (Ile-de-France, the Paris metropolitan region) who were younger than 15 years when diagnosed with a histologically-confirmed medulloblastoma from 1990 through 2005. The study has been described in detail elsewhere [25]. The geographic exhaustiveness of the recruitment was verified from the French National Registry of Childhood Solid Tumors [29], [30].

### Data collected

We collected the following data from each medical file in the neurosurgery and oncology departments: age, sex, symptoms, the radiologic and pathology characteristics of the tumor (standard or nodular/desmoplastic histology [31], [32], the latter referred to hereafter as desmoplastic), local and metastatic staging, postoperative status (posterior fossa syndrome), vital status and neurological outcome at last follow-up (full scale intelligence quotient (IQ) score and neurological examination, classified into 3 groups: strictly normal neurological examination; moderate and unilateral dysmetria without functional consequence; or neurological disability). Pathologists at each hospital (sites of the neurosurgery and oncology departments) confirmed all histological diagnoses of biopsy or resection samples within days of the radiological diagnosis. The time to diagnosis, expressed in days, was defined as the interval between the first symptom attributable to the disease and the date of diagnosis (date of brain imaging). When ambiguous (for 2% of patients), it was independently evaluated by 3 of the authors, who reached a consensus.

Tumor stage was assessed: (1) by cerebral MRI, before and shortly after surgery, to determine the completeness of the resection, (2) pre- or postoperative spinal MRI, and (3) a postoperative lumbar puncture to look for metastasis. All patients had a complete staging work-up, except one who died within 24 hours of surgery. Based on the surgical report and early postoperative imaging, the initial disease stage was classified by the T stage according to the Chang-Harisiadis classification [33], [34] and according to the classification of risk groups recognized by the International Society of Pediatric Oncology: standard risk (complete resection), local high risk (incomplete resection), and metastatic high risk [11]. The tumor volume was calculated according to the formula for the volume of an ellipsoid: 4π/3×(height×width×depth)/8. During the study period, patients were treated with surgery, chemotherapy, and radiotherapy according to the national or European protocols [3]–[5], [35]–[37].

### Statistical analyses

First, we described the demographic and tumor characteristics and the time to diagnosis. Second, we studied the relation between this prediagnosis interval and initial severity factors (metastasis, tumor volume, T stage and completeness of resection) by univariate (Kruskal-Wallis test) and multivariate analysis, taking into consideration the cofactors of interest, by either stratification or adjustment in a backward stepwise logistic regression model. Third, we studied the relations between time to diagnosis, survival, and neurological and neuropsychological outcome. These analyses were performed first for the entire population (Kaplan-Meier method and logrank test), then after stratification for the identified prognostic factors, and finally by adjustment for the cofactors of interest in a Cox model. One of the Cox models was constructed by adjusting the relation between time to diagnosis and survival by a propensity score for a long prediagnosis interval [38], calculated by the logistic regression equation produced at stage 2. The time to diagnosis was used as a binary variable after dichotomization around the median or as a continuous variable (after testing linearity). Age was dichotomized around 5 years, in view of the difference in treatment around this age [4], [5]. Statistical analyses were performed with STATA (StataCorp.).

### Ethics Statement

The Institutional Review Committee (Comité de Protection des Personnes Ile-de-France III) stated that “this research was found to conform to scientific principles and ethical research standards and to the laws and regulations of France” and specifically approved this study. Written informed consent of the patients or their parents was not judged necessary for this kind of retrospective study. Data were anonymized before the clinical records were included.

## Results

### Population, distribution of time to diagnosis

Examination of the diagnostic code lists showed 170 patients eligible for this study. Of these 170 eligible files, 4 (2%) were lost or incomplete. The analysis therefore concerned 166 patients, 72% of whom were boys. Median age at the first symptom was 6 years (interquartile range (IQR): 4–9) and 31% were younger than 5 years.

The patients had a local tumor in 62% (standard risk 40%, local high risk 22%) and a metastatic tumor in 38% of cases. The median tumor volume was 33 cm^3^ (IQR 22–42). Staging showed 2% of the children had T1 local tumors, 27% T2, 15% T3A, 39% T3B, and 17% T4. The surgeon observed fourth ventricle floor invasion in 47% of the 161 cases for which surgery was performed. Resection was complete in 52% of cases. The histologic type was standard for 78%, desmoplastic for 22%, and large-cell anaplastic for one patient (pooled with the “standard histology” patients for subsequent analysis).

Time to diagnosis was determined in all cases. Its median was 65 days (IQR 31–121, range 3–457). We have previously described its distribution in detail [25]. After dichotomization (table 1), a prediagnosis interval greater than the median of 65 days (hereafter, a long interval or time to diagnosis) was associated with a standard histologic type (vs desmoplastic) and with psychological symptoms (such as impaired school performance, behavioral problems, depression, or anxiety). The patients younger than 5 years at diagnosis had a significantly shorter interval (median 55 vs 77 days, *p* = 0.03), but after dichotomization (table 1), an interval greater than the median of 65 days was not statistically associated with an age older than 5 years (*p* = 0.13).

**Table 1:** Relation between time to diagnosis (TtD) and clinical and tumor characteristics.

				short TtD	long TtD				
Characteristics	*n*	median TtD	*p* §	<65 days	>65 days	OR [95% CI]¤	*p* ±	ORa [95% CI]¤¤	*p* ‡
	(total = 166)	(days)		(n = 83)	(n = 83)				
**Age at diagnosis** <5 years	51	55	0.03	30	21	1.7 [0.8, 3.4]	0.13	1.3 [0.6, 2.7]	0.5
>5 years	115	77		53	62				
**Psychological signs** **: impaired school performance, depression, behavioral problems, anxiety									
absent	122	60	0.001	68	54	2.4 [1.1, 5.3]	0.01	2.5 [1.1, 5.6]	0.03
present	44	91		15	29				
**Tumor characteristics**									
standard histology	129	61	0.01	70	59	2.4 [1.03, 5.5]	0.03	2 [0.8, 4.7]	0.12
desmoplastic	36	112		12	24				
metastatic tumor	62	31	<10^−4^	49	13	8 [3.6, 18]	<10^−6^	7.6 [3.6, 16.4]	<10^−3^
local tumor	103	91		33	70				
missing data*	1			1	0				

§ Degree of significance of nonparametric test (Mann Whitney or Kruskal-Wallis) of the distribution of time to diagnosis.

¤ Odd Ratio [95% confidence interval].

± Degree of significance of the chi-2 test or Fisher's exact test.

¤¤ adjusted Odds Ratio [95% confidence interval].

‡ Degree of significance of the coefficient of the logistic regression test.

* One patient died within 24 h of surgery, before spinal MRI, and was excluded from the analysis.

** The data concerning psychological signs have been previously published [25].

### Relations between time to diagnosis, tumor stage, and cofactors

**Figure 1:**
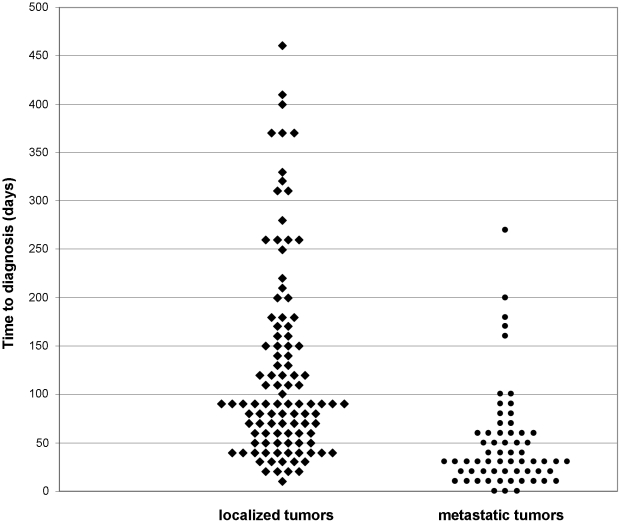
Distribution of time to diagnosis according to the presence of metastatic disease (*p*<10^−4^).

The children with metastatic disease had a significantly shorter prediagnosis interval than those with local tumors (median 31 vs 91 days, p<10^−4^; figure 1, table 1). Overall, tumor volume was significantly larger for the patients with a long time to diagnosis (median tumor volume 34 vs 27 cm^3^, *p* = 0.002; table 2). We did not find a statistically significant (p>0.2) association between time to diagnosis and any of the other factors related to local extension (T stage and fourth ventricle floor invasion) or completeness of surgical resection.

**Table 2:** Relation between time to diagnosis (TtD) and local extension.

	all patients			metastatic tumors			local tumors		
Local extension	*n*	median TtD	*p* *	*n*	median TtD	*p* *	*n*	median TtD	*p* *
	(total = 166)	(days)		(total = 62)	(days)		(total = 103)	(days)	
**tumor volume**									
small (<1^st^ tercile)	55	45	0.002	20	23	0.005	34	71	0.10
intermediate (2^nd^ to 3^rd^ tercile)	55	69		21	33		34	118	
large (>3^rd^ tercile)	56	86		21	49		35	94	
**stage of local extension** ‡									
T 1 or T2	49	75	0.35	12	13	0.01	36	139	0.15
T 3A	25	62		8	27		17	76	
T 3B	64	61		34	41		30	80	
T 4	28	84		8	45		20	95	
**infiltration of the 4^th^ ventricle floor**									
not invaded	85	68	0.37	20	24	0.006	64	92	0.29
invaded	76	62		38	44		38	87	
no surgery	5	41		4	29		1	96	
**tumor resection**									
complete (according to surgeon and imaging)	86	65	0.43	19	26	0.05	66	92	0.72
incomplete	75	69		39	41		36	89	
no surgery	5	41		4	29		1	96	

* Degree of significance of nonparametric test (Mann-Whitney or Kruskal-Wallis) of the distribution of time to diagnosis.

‡ Local extension stages according to Chang-Harisiadis classification [33], [34].

For the 103 patients (62%) without metastatic disease (table 2), we did not find a significant association between time to diagnosis and local extension (volume, T stage, or fourth ventricle floor infiltration). Similarly there was no association with the completeness of surgical resection among the 102 who had surgery (that is, prediagnosis interval did not differ for standard risk and high-risk local tumors: 89 vs 92 days, p>0.2). For the 62 patients (38%) with metastatic disease, local extension was significantly greater for long prediagnosis intervals: the tumor volume was larger, the T stage more advanced, and fourth ventricle floor infiltration more frequent (table 2). Of the 58 patients with metastatic disease who had surgery, the median prediagnosis interval was 26 days for those with complete resection and 41 days for incomplete resection (*p* = 0.05).

After adjustment by logistic regression for all of the potentially explanatory factors associated with a long prediagnosis interval in the univariate analysis with a p value<0.2 (i.e., age older or younger than 5 years, psychological symptoms, histologic type, and metastasis), two factors were significantly and independently associated with an interval longer than the median: psychological symptoms (adjusted odds ratio (ORa) = 2.5 [1.1–5.6]; *p* = 0.03) and the absence of metastasis (ORa = 7.6 [3.6–16.4]; p<10^−3^). The associations with age and histologic type were not statistically significant after adjustment (table 1). The four variables above and the exact coefficients of the logistic regression equation were used to construct the propensity score for long time to diagnosis used below.

### Relations between time to diagnosis, survival, and cofactors

**Figure 2:**
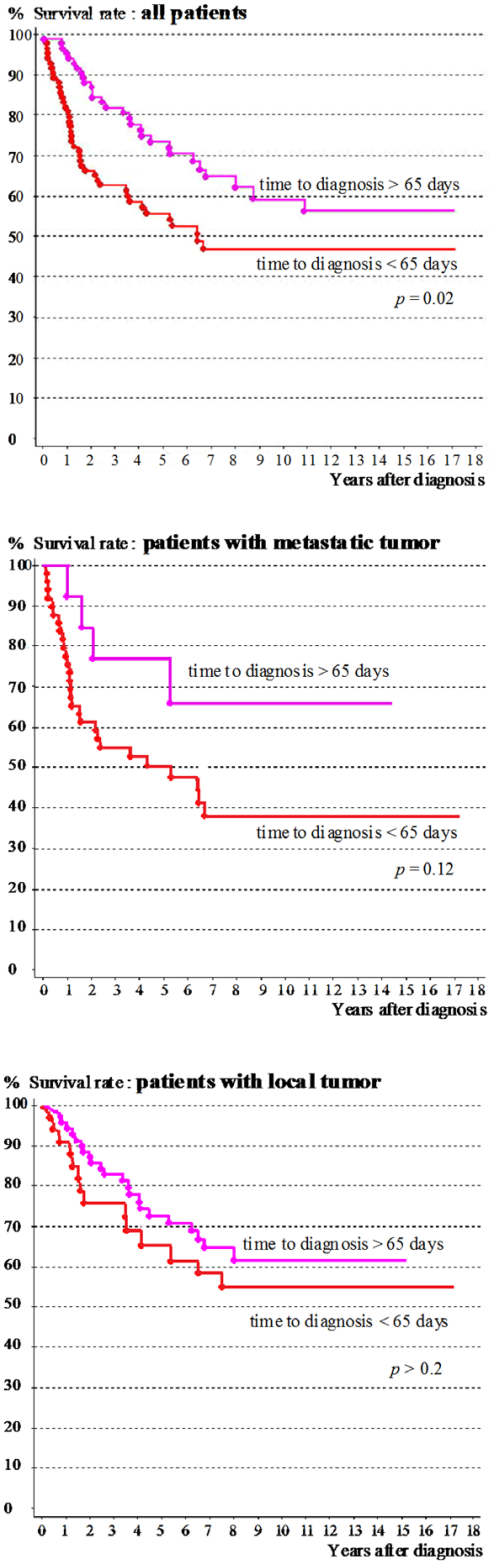
Survival according to time to diagnosis (more or less than the median of 65 days).

No patients were lost to follow-up. At the last follow-up, 96 patients (58%) were still alive. The median follow-up was 7 years (IQR 5–12 years, range 3–17 years). All survival rates reported below are 10-year rates. The survival of patients with a long time to diagnosis was significantly better than that of patients with a short prediagnostic interval (60 vs 47%, relative risk (RR) = 1.8 [1.2, 2.8], *p* = 0.02; figure 2). Survival rates were significantly better for patients older than 5 years, those without metastatic disease, those with desmoplastic tumors and those with complete tumor resection (table 3).

**Table 3:** Survival according to age, tumor characteristics, and time to diagnosis (TtD).

			relative risk		adjusted relative risk	
Characteristics	*n*	10-year survival	[95% CI]	*p* *	[95% CI]	*p* *
	(total = 166)	(%)	univariate analysis		multivariate analysis‡	
TtD<median (65 days)	83	47	1.8 [1.2, 2.8]	0.02	1.5 [0.8, 2.5]	0.17
≥median	83	60				
Age at diagnosis <5 years	51	45	1.9 [1.2, 3.1]	0.007	1.7 [1.0, 2.8]	0.04
>5 years	115	57				
Metastatic tumor§	62	43	1.8 [1.1, 2.9]	0.01	1.3 [0.7, 2.2]	0.4
Localized tumor	103	59				
Standard histology	130	48	2.1 [1.1, 4.1]	0.03	1.7 [0.9, 3.4]	0.12
Desmoplastic histology	36	72				
Incomplete resection or no surgery	80	44	1.7 [1.1, 2 .7]	0.03	1.6 [0.9, 2.6]	0.09
Complete tumor resection	86	68				
Tumor volume>median (33 cm^3^)	83	53	1.1 [0.7, 1.8]	0.6	-	-
<median	83	54				

* Degree of significance of the Logrank test.

‡ Cox model with adjustment for the following covariables: TtD less than or more than the median of 65 days, age older or younger than 5 years, desmoplastic or not histology, metastatic or localized tumor.

§ One patient died within 24 h of surgery, before spinal MRI, and was thus excluded from the analysis.

After stratification by age, survival rates for the children younger than 5 years were best for those with a long time to diagnosis (67 vs 29%, RR = 3.6 [1.4, 8.9], *p* = 0.03); for those older than 5 years, survival did not differ significantly according to the length of this interval (57 vs 56%, RR = 1.1 [0.6, 2.1], p>0.2). Stratification by metastatic disease showed that survival for the patients with metastatic tumors was 66% among those with a long interval vs 38% for those with shorter ones, but this difference was not statistically significant (RR = 2.2 [0.8, 6.4], *p* = 0.12); for patients without metastatic disease, survival did not differ significantly according to prediagnosis interval (62 vs 55%, RR = 1.1 [0.6, 2.3], p>0.2). After stratification by histologic type, survival was better in the group with standard tumor histology when their time to diagnosis was longer than the median (55 vs 42%, RR = 1.8 [1.1, 3.1], *p* = 0.02); in the group with a desmoplastic tumor (36 patients), survival did not differ significantly according to this duration (72 vs 71%, RR = 1 [0.3, 3.7], p>0.2). After stratification by completeness of the resection, survival among the patients with incomplete resection or no surgery was better for those with a long interval (54 vs 36%, RR = 2.0 [1.1, 3.8], *p* = 0.02); for the patients with complete resection, survival did not differ significantly according to time to diagnosis (65 vs 57%, RR = 1.5 [0.73, 3.1], p>0.2).

After adjustment in a Cox model for the covariables of interest associated with survival in the univariate analysis with a p value<0.20 (i.e., time to diagnosis, age, metastasis, histology, and complete resection), only age older than 5 years was independently associated with survival (adjusted relative risk (RRa) = 1.7 [1.0, 2.8], *p* = 0.04). Survival was not significantly associated with time to diagnosis, metastasis, complete resection, or histologic type (table 3). In two other models, supplementary adjustment for the use of radiotherapy (upfront, delayed or omitted in some children under 5), or for the propensity score did not significantly change the relation between time to diagnosis and survival (RRa = 1.6 [0.9, 2.8], p = 0.10 and RRa = 1.5 [0.8, 2.5], p = 0.17, respectively). Using time to diagnosis as a continuous variable did not significantly change the results either.

### Relations between time to diagnosis, IQ score, and neurological disability

Postoperative posterior fossa syndrome was described for 23% of the patients. The neurological examination of the 96 survivors was strictly normal for 30% of patients, showed moderate and unilateral dysmetria without functional consequence for 36%, and neurological disability for 33%. The median IQ score for the 80 (83%) of the 96 survivors for whom the IQ score was available was 78 (IQR: 68–94; range 42–131).

**Figure 3:**
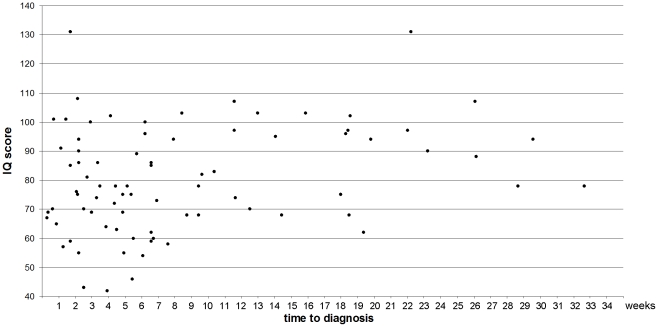
IQ score according to time to diagnosis (p<0.05, see text).

The presence of postoperative posterior fossa syndrome was associated with a significantly shorter time to diagnosis (median 50 vs 75 days, p = 0.02). The time to diagnosis was not significantly different (p>0.2) between patients with normal neurological findings, with moderate and unilateral dysmetria without functional consequence, or with neurological disability (median 87 vs 72 vs 72 days, respectively). The IQ score was significantly associated with the prediagnosis interval, both after linear regression (figure 3, p = 0.01), following transformation with a fractional polynomial, and after adjustment for age, radiotherapy dose, and the covariables mentioned above (p<0.05).

## Discussion

### Main results

We found complex and often inverse relations between a longer time to diagnosis of medulloblastoma in children, the initial severity factors, and survival. A long prediagnosis interval was associated with a larger tumor volume, a lower frequency of metastasis, desmoplastic histology, and longer survival in the univariate analysis but not after adjustment. The time to diagnosis was significantly associated with IQ score among survivors. No significant relation was found between the time to diagnosis and neurological disability. In the 62 patients with metastatic disease, a long prediagnosis interval was associated with a more advanced T stage, fourth ventricle floor invasion, and incomplete surgical resection; it nonetheless did not influence survival significantly in this subgroup.

### Hypothesis

The inverse relation between time to diagnosis and severity of disease may be explained by the type of tumor progression. Rapidly growing and metastatic tumors might produce swift and intense clinical signs, leading to rapid consultation and diagnosis, but for a very advanced tumor. Inversely, local tumors that grow slowly might cause relatively mild and very progressive clinical signs that would lead to a long period of development before diagnosis. Recent studies of patients with medulloblastoma indicate that outcome is determined much more by underlying molecular biology [39] than clinical factors such as time to diagnosis.

### Study limitations

Our population-based cohort allowed us to avoid the recruitment bias of single-center studies. The principal limitation of the study is its retrospective nature. Nonetheless the data came from multiple sources and the time to diagnosis was ambiguous for only 2% of patients. Disease extension was determined in all but one patient (<1%), based on standardized measurement methods (imaging). No patients were lost to follow-up. Most studies of time to diagnosis are retrospective, which may lead to some bias, but it is non-differential bias.

Our results are consistent with those of a previous study about the relation between short prediagnostic intervals and metastasis [13]: we confirmed their results in this more recent, larger and exclusively pediatric population-based study that took confounding factors into account and analyzed the consequences for initial tumor stage and survival. Finally, we found the same demographic, clinical, tumor, and prognostic characteristics as in other series of medulloblastoma in children [9]–[15], [40].

### Ethical and legal issues

The results of our study suggest that the time to diagnosis of medulloblastoma is related more to the properties of the tumor than to suboptimal care by either parents or healthcare personnel. This information should reassure parents who feel guilty about the delay until the first consultation for symptoms finally attributed to the medulloblastoma, a delay sometimes attributable to their psychological or banal nature [23], [25]. Parents, in their quest to determine the origin of the disease, feel remorse and guilt for having neglected the initial symptoms, especially when the disease outcome is unfavorable [26]. The diagnosis delay associated with the physician is equally the source of painful regret [26] as well as lawsuits [28]. Our study suggests that there is no simple causal relation between time to diagnosis and harm or damage, even in cases of metastasis, incomplete resection, or large tumor volume. This finding is highly pertinent in a medical malpractice system based on tort law [28], even if the complaint alleges specific acts, of negligence for example, by the doctor rather than the length of the delay alone. Moreover, the length of the diagnosis delay depends on two separate factors: the patient delay, that is, the time interval between the onset of symptoms and the first presentation to a healthcare professional, and the doctor delay, that is, the time interval between the first medical consultation and the final diagnosis. In our study, the median duration of the latter was 30 days and accounted for slightly under half (46%) of the total delay. However, because these data were available for only 40% of the files, they were not analyzed in detail. Finally, the conclusions about the individual consequences of a given diagnosis delay may well differ from the conclusions drawn from the analysis of a cohort of patients.

### Conclusion

Although a longer time to diagnosis may not be related to inferior prognosis, we cannot and do not claim that a “longer than necessary” time to diagnosis will not lead to high-risk disease, need for more intensive therapy, and possibly a worse outcome. The inverse association between time to diagnosis and prognosis in a group of patients does not mean that a delay in diagnosis can have no consequences for an individual patient. Moreover, a rapid diagnosis shortens children's suffering and helps prevent parents' loss of confidence in the health care system [26].
